# Placebo effects in allergen immunotherapy: an experts’ opinion

**DOI:** 10.1007/s40629-018-0065-z

**Published:** 2018-05-24

**Authors:** Anthony J. Frew, Oliver Pfaar

**Affiliations:** 1 Department of Respiratory Medicine, Royal Sussex County Hospital, Brighton, UK; 2 Department of Otorhinolaryngology, Head and Neck Surgery, Universitätsmedizin Mannheim, Medical Faculty Mannheim, Heidelberg University, Mannheim, Germany; 3 Center for Rhinology and Allergology, Wiesbaden, Germany; 4 Brighton & Sussex Medical School, Royal Sussex County Hospital, University of Sussex and University of Brighton, BN2 5BE Brighton, UK

**Keywords:** Sublingual, Subcutaneous, Clinical trials, Endpoints, EAACI Task Force

## Abstract

Placebo effects are common in medicine. Randomised clinical trials help us to understand their magnitude in different therapies. There are particular problems with placebo effects in allergen immunotherapy (AIT) as it is difficult to blind the active treatment and the endpoints are largely subjective. This may explain why large placebo effects are often found in AIT trials. Patients receiving open label AIT get the benefit of the active and placebo components but it can be difficult to say how much benefit is due to the active component. The use of active placebos has been proposed but brings its own problems (ethical and scientific). An EAACI Task Force has been established to address these issues. Here we review the current literature on the placebo effect in general, with a special focus on AIT trials, and indicate what we believe to be important considerations and unmet needs in AIT trial design.

## Introduction

Placebo effects are common in medicine and the underlying complex neuropharmacological and -anatomical mechanisms involved have mainly been investigated in the field of pain and Alzheimer’s disease [1]. Moreover, placebo effects play a major role in allergic diseases which also have been demonstrated to be much affected by this phenomenon [2]. Especially in trials on allergen immunotherapy (AIT) by both the sublingual (SLIT) and subcutaneous (SCIT) route, placebo effects of large magnitudes have been reported and several hypotheses for these effects have been suggested [3, 4]. Those factors undoubtedly have an important bearing on the design, execution and interpretation of results of clinical trials in AIT. The purpose of a current Task Force (TF) initiative therefore is to review this phenomenon with a number of stakeholders in the field of AIT comprising clinicians, methodologists, regulatory and patient representatives and to develop an EAACI Position Paper on this topic. The following review overviews the current literature on the placebo effect with a special focus on AIT trials, but also indicates important considerations and unmet needs in the view of experts’ opinions.

## Psychological and sociological mechanisms of the placebo effect

The placebo effect has been known for many years and goes right back to the writings of Hippocrates. Literally, “placebo” means “I will please”, and encapsulates the psychological and physical benefits of seeking advice and treatment for a problem, independent of the pharmacological effects of the prescribed therapy [1]. There is therapeutic value in simply talking to someone about your problem, which helps you to debrief and put the problem into perspective. We are social animals: sympathy from a friend (or a physician), and sharing our problems is a deep-seated human instinct and is psychologically helpful. Empathy—the sense that someone else cares about you, and that your concerns are both real and matter to someone else—can be therapeutic. Reassurance that you are not alone helps people to cope. And then sometimes a treatment is offered which may or may not work, but the whole process of problem sharing and then obtaining advice and treatment is all rolled into the perceived value of each medical consultation [1].

Many diseases, including allergic conditions, show natural variation over time. One of the reasons humans have been successful is that we are good at making connections between events and possible causes. This is useful and helps us to develop strategies for reacting to similar events in the future. However, it also leads to false attributions of cause and effect. In crude terms our brains take “I did X and Y happened” and we conclude “Y happened because I did X”. And then we continue to do X in the hope that Y will happen, and it can take quite a long time before we find that the two events are in fact unconnected, especially if the action and desired effect are turned into a form of superstition or a formal religious belief.

So in our clinical practices we often meet people who have fluctuating conditions, like urticaria and irritable bowel syndrome, who incorrectly attribute flares of their condition to food allergy. But the ability to structure their diet and feel that they can control their condition may be useful, as it gives them back a sense of control, which is psychologically beneficial. Similarly, if a condition fluctuates randomly it is more likely that you will seek advice when the condition is flaring up, and then as the condition spontaneously improves, you may attribute that improvement to any treatment you have just received. If you always take the same treatment (which actually does nothing) whenever the condition flares up and then you spontaneously get better, you will come to believe that the treatment is actually driving the improvement.

## Relevance for study designs in the age of evidence-based medicine

Science is meant to be the way that we explore these phenomena and find out whether there really is a causal relationship. Science works by disproving things—a successful experiment is one where you design a test which shows convincingly that two things that you thought were connected are in fact only occurring together randomly. Unfortunately, in medicine we tend to like studies that confirm our prejudices. We talk about “positive results” and “experiments that worked” when we find a connection, when in fact an experiment only really works when it disproves the hypothesis [5]. And when it comes to marketing drugs and treatments, there is a natural tendency for companies to promote data which supports their marketing strategy, rather than data which casts doubt on the product’s efficacy. This “publication and presentation bias” has the unintended but entirely predictable effect of making us feel that our treatments are more effective than they truly are.

To address this we need clinical trials—these help us to assess the true effectiveness of interventions in terms of who benefits, how much they benefit by, and which outcomes are affected. Trials also allow us to assess the harmful potential of interventions and they lead on to decisions on licensing and whether to fund treatments through insurance and state-funded healthcare systems [5]. In some cases the trials show little or no effect compared to a control group. The history of medicine is littered with treatments for which those with enthusiasm have no controls, while on the other hand those with controls have no enthusiasm.

Double-blind placebo-controlled clinical trials (DBPCCT) are the gold standard for assessing the effectiveness of therapies [5, 6]. The main aim of a DBPCCT is to reduce the scope for bias due to physicians choosing who gets active treatment, bias in reporting outcomes (by the participant and the observer) and bias in reporting (or ignoring) side-effects. But there is a very real question whether the criteria for a DBPCCT can be met for trials of allergen immunotherapy (AIT).

To perform a DBPCCT properly, you need to have patient groups that are randomly allocated to receive active and control therapy, with blinding of allocation, intervention, observation of outcomes, and analysis of data. You also need a sufficient level of symptoms or endpoints in the control group to avoid the possibility of missing an effect due to lack of signal (i.e. insufficient room to see a reduction in the chosen endpoints). This can partly be addressed by a power calculation, but that presumes that you can estimate the likely effect size. The power of a study is also affected by any heterogeneity of response, in terms of both proportion and scale—the greater the heterogeneity of response, the lower the power of a study to show any effect [5].

The robustness of trial endpoints varies between different clinical areas. Some endpoints are relatively immune from subjective influences. So death is a robust endpoint—it either happens or it doesn’t. Measurements of blood cholesterol or renal function are relatively robust—once the sample is taken, the lab guarantees that the measurement is accurate. Blood pressure is slightly less robust and can be affected by suggestions from the observer, as well as by the beliefs of the patient. Other parameters used in clinical trials are much more open to subjective influences, including symptom scores, medication scores, measures of quality of life (QoL) and physicians’ global assessment which are all dependent on the subject and the investigator applying their judgement to score and grade their internal feelings [6].

## Placebo effects in AIT

In his seminal paper on subcutaneous AIT (SCIT), published in 1914, John Freeman noted that SIT led to “a distinct amelioration of symptoms” and cautioned that “there is a constant tendency to detect such improvement in adventitious fluctuations of health.” He also noted that “a patient inoculated with one pollen became immune to another”, but he was not clear whether this was a true bystander effect or an indirect consequence of people having less symptoms during the pollen season and therefore becoming more tolerant of other exposures [7].

The first randomised clinical trial of SCIT was conducted by Frankland and Augustin in the early 1950s. They did a controlled trial comparing crude grass-pollen extracts with the isolated main protein component in the prophylaxis of summer hay-fever and asthma [8]. Ten years later, Lowell et al. published the first North American RCT of SCIT [9]. They matched patients on multiple SCIT in pairs in terms of their symptoms in the ragweed season and then removed the ragweed from one of each pair’s vaccine and replaced it with caramelised sugar solution. The study was reasonably blinded as patients still had local reactions due to the other components of their vaccines. By this method they confirmed the allergen-specificity of AIT and that you needed to receive ragweed in your vaccine to get protection against natural exposure to ragweed.

There are some issues in conducting DBPCCT of AIT that are specific to this treatment. Unlike most of the tablets tested in clinical trials, AIT injections can elicit local and systemic reactions. This makes it difficult to blind subjects to which treatment they are receiving. Most of those who receive active therapy will have some local side-effects during the trial and, although you can ask patients which treatment they are receiving, it is indisputable that experiencing side-effects is likely to impact on the perception of symptoms. This in turn may lead those in the active group to report their symptoms differently. Those who do not get side-effects and think they are in the control group may be disappointed not to have received active treatment and the supervising trial staff may pick up who had active treatment and who did not. It is not necessary for all participants to be aware of which arm they are in for the integrity of the DBPCCT to be affected. With sublingual immunotherapy (SLIT), where the allergen is applied to the oral mucous membrane, local side-effects are extremely common, affecting over half of the patients receiving active treatment [10], which further complicates our ability to achieve blinding of allocation in clinical trials of SLIT. Another important bias in AIT trials is the level of motivation of patients to participate and to benefit from treatment [11].

With these caveats, clinical trials of AIT do demonstrate efficacy—with clear differences between the experience of active and control groups even in the first season of grass pollen AIT [12, 13]. However, these parallel group trials do not tell us how much of a placebo effect may be operating. Other trials with run-in seasons have shown very large placebo effects—for example a 2-year trial of SLIT in children aged 6–14 years defined improvement as a decrease of >30% in symptom scores. This was achieved in 85% of the active group but also in more than half of the placebo group, but there was no effect on medication use, which serves as a surrogate marker of efficacy [14]. Other clinical trials of SCIT also designed with an initial run in (baseline) period have demonstrated placebo effects of 6 to 52% for different extracts (reviewed in [4]).

Conversely, a clinical trial of AIT for cat allergy found very stable thresholds for conjunctival provocation tests in those who received placebo treatment while the actively treated group showed a dramatic reduction in sensitivity [15]. The paediatric SLIT study mentioned above showed a strong placebo effect on CPT thresholds after one year of treatment but by the time of the final assessment after two years treatment, the placebo effect had disappeared [14]. Another interesting example of a huge placebo effect are data recently published on CAT-SPIRE (synthetic peptide immuno-regulatory epitopes) for SCIT in cat allergic patients (CATALYST; [16]). Though results from a preliminary phase II (dose–range finding) trial performed in an allergen exposure chamber were very promising [17], the subsequent field (pivotal) trial failed in demonstrating a meaningful clinical additional treatment effect to the placebo effect itself which already improved patients’ symptoms by approximately 60% [16]. Explanations for this high placebo effect may be seen in the high motivation of the participating patients, a possible disadvantage of inflated baseline symptoms or spontaneous remission of symptoms (“natural desensitization” under daily allergen exposure), but these should be further investigated and predictors of a high placebo response evaluated [1, 11, 16, 18].

A Cochrane review of SLIT trials, which confirmed the efficacy of SLIT in a systematic assessment of SLIT studies, commented that there were some limitations to applying meta-analysis techniques to the older studies because many of the early SLIT studies were rather small, with variable dosage, variable endpoints and limited baseline data, so often the active and placebo groups were ill-matched. But taking everything into consideration, there were large placebo effects in most published studies of SLIT [19], whereas another analysis has controversially found only a minimal impact of the placebo effect in SLIT trials [4].

## Challenges and considerations for the future

Nearly 20 years ago, reviewing the field, we argued that the time for small studies was over, and that what we needed were large randomised DBPC studies, with carefully balanced groups of subjects, whose disease was moderately severe and whose sensitivity to the target allergen was clearly defined. As well as prolonged run-in periods collecting of baseline data to harmonise the groups before allocation to treatment, we proposed that better, more valid clinical endpoints were needed together with an economic evaluation and careful documentation of side-effects. And ideally the trials should be conducted in a less intensive setting, more typical of where the treatment would be deployed as compared to the usual clinical trials unit [20].

But even when that strategy was followed, significant placebo effects can be seen in AIT studies [4, 21]. While it is possible to make statistical adjustments in order to look at the proportion of subjects who improve more than average, or to back up the symptom data with medication scores [6], the fundamental problem remains of side-effects potentially affecting the blinding of the trial. Similar considerations apply to studies using patients’ or physicians’ global assessments of efficacy. Because a substantial excess of subjects receiving placebo report overall improvement, the scope for showing an effect of active therapy is reduced. Of course, if there is a true placebo effect, then once the drug is used in open label studies, the recipients get both the true effect and the placebo benefit, so the patients benefit from any placebo effect, even if the funding body may be getting less true benefit per euro expended.

On the observation side, the main problem for DBPCCT design is the subjectivity of the symptom and treatment scores. The lack of agreed objective endpoints or measures of success contributes to heterogeneity of studies but also makes us more dependent on subjective parameters, as compared to other areas of medicine. Analysis of the data is not helped by the non-parametric nature of most scoring systems which use ordinal numbers (0,1,2,3) and then add them and average them as if they were parametric variables, with a single step from 0–1, 1–2, or 2–3 equivalent in terms of clinical relevance. The frequency of visits and the interest of study team add to the potential for taking part in the trial to lead to an improvement in symptoms, without there being any therapeutic benefit from the treatment.

So, in summary, the endpoints for SCIT and SLIT trials are largely subjective [6, 22], which is not a problem in itself, but becomes an important issue when the basic criteria for DBPCCT are not met, especially when there is difficulty concealing allocation to the active and placebo groups. Although not universally true, subjects often know which arm of the trial they are in, and those making observations are also likely to pick this up, which introduces potential bias in the assessment process. Some endpoints which are superficially objective, like conjunctival provocation tests, may also be affected if people know which arm they are in. Remedies for this problem have been difficult to agree: the inclusion of histamine in the placebo treatment is one option, but that predictably causes side-effects whereas the active treatment causes random side effects and moreover, there is good evidence that histamine itself is immunomodulatory, through the presence of histamine receptors on the surface of T‑lymphocytes [23].

## Conclusions

Placebo effects play a fundamental role in the magnitude of efficacy of treatment options in all diseases. In the field of allergen immunotherapy, several factors impact this effect which should be taken into account when planning, performing and analysing clinical trials. This review gives a current overview of possible roles of different mechanisms affecting the placebo response in AIT and discusses challenges and unmet needs. These and other related issues are the subject of a current EAACI Task Force initiative which aims to report consensus-based definitions and recommendations in a Position Paper.

## Abbreviations

AIT: Allergen immunotherapy
DBPC: Double-blind, placebo-controlled
EAACI: European Academy of Allergy and Clinical Immunology
QoL: Quality of life
SCIT: Subcutaneous immunotherapy
SLIT: Sublingual immunotherapy
SPIRE: Synthetic peptide immunoregulatory epitopes
TF: Task Force
